# Alveolar bone loss and tooth loss contribute to increase in cancer mortality among older patients

**DOI:** 10.1186/s12903-023-03543-5

**Published:** 2023-12-19

**Authors:** Yifeng Qian, Binxin Cai, Fangfang Chi, Chunxia Yao, Lei Zhang, Lei Qi, Yonggen Jiang, Xudong Wang

**Affiliations:** 1 Department of Oral and Craniomaxillofacial Surgery, Shanghai Ninth People’s Hospital, College of Stomatology, Shanghai JiaoTong University School of Medicine, 639 Zhizaoju Road, Huangpu District, Shanghai, China; 2 National Clinical Research Center for Oral Diseases, Shanghai, China; 3 Shanghai Key Laboratory of Stomatology & Shanghai Research Institute of Stomatology, Shanghai, China; 4 College of Stomatology, Shanghai JiaoTong University School of Medicine, Shanghai, China; 5 Songjiang District Center for Disease Control and Prevention, Shanghai, China

**Keywords:** Cancer risk, Tooth loss, Alveolar bone loss, Mortality

## Abstract

**Background:**

Both cancer and periodontitis are more prevalent with age. Information on their relationship in older patients is limited. This study aims to examine whether periodontitis is associated with increased risk of cancer mortality with a ≥ 75-year age group cohort.

**Methods:**

A retrospective cohort study was conducted on 1146 patients who had digital radiographic examinations. Alveolar bone loss and loss of teeth were measured as indicators of periodontitis. Hazard ratio (HR) with 95% confidence interval (CI) were taken as the effect size to summarize the associations between periodontitis and risks of cancer mortality using the multivariate adjusted cox proportional hazards model and competing risk hazard model.

**Results:**

Totally, 104 total cancer, 28 lip, oral cavity and pharynx (LOP) cancer, 39 digestive cancer and 13 respiratory cancer cases were documented over 10 years of follow-up. Total cancer (HR 1.27, 95% CI 1.06–1.53) displayed statistically significant associations with alveolar bone loss and tooth loss after adjusting for relevant confounding variables. We also observed borderline significant association between alveolar bone loss and LOP cancer (HR 1.45, 95% CI 0.99–2.12). The above associations were consistent with the results observed from the competing risk hazard models.

**Conclusion:**

Our results indicate that older patients suffering from tooth loss or alveolar bone loss are at increased risks of cancer mortality, especially for total cancer and LOP cancer.

**Supplementary Information:**

The online version contains supplementary material available at 10.1186/s12903-023-03543-5.

## Introduction

Globally, 11% of the population suffer from severe periodontitis, with an age-standardized incidence rate of 701 per 100,000 person-years [1]. Periodontitis, a chronic inflammatory disease, results from bacterial infection of gingival and bone tissues around the teeth [2]. Tooth loss and edentulism are common consequences of severe periodontitis [3]. Researchers have found associations between periodontitis and cerebrovascular, coronary heart, and pulmonary diseases because of possible shared factors [4–7]. In recent years, it has become increasingly important to understand the relationship between this inflammatory condition and several malignancies, such as oral, lung, and prostate cancer [8–12].

The global population is undergoing a significant demographic shift, with the proportion of older people projected to increase at a faster rate than any other age group in the subsequent years. It is particularly important to assess periodontitis and cancer risk among older subjects owing to the increased prevalence of periodontitis with age and the long latency period of most cancers [13].

Inflammation that enters the bloodstream, pathogen invasion into the bloodstream, and immunosuppression are possible mechanisms by which periodontitis increases the cancer risk [14, 15]. In this retrospective cohort study, loss of tooth and alveolar bone loss, measured as indicators of periodontitis, and the risk of cancer mortality were assessed, after taking into account potential confounding factors.

## Materials and methods

During 2010–2014, 30,169 individuals aged ≥ 75 years visited Shanghai Ninth People’s Hospital, the largest dental hospital in Shanghai, and received periodontal treatment. We conducted a case-by-case inquiry to obtain imaging data, ultimately including 1146 patients with comprehensive imaging data, specifically panoramic radiographs, and no previous history of cancer.

### Dental examination

The radiograph for each patient was divided into four quadrants. Measurement of the tooth with the most severe alveolar bone resorption in each quadrants was made from the cementoenamel junction to the tooth apex and from the marginal bone crest to the tooth apex. We calculated the proportion of the remaining bone height from the total root length and total bone height. Then the above proportions were averaged and each patient was classified as healthy (≥ 0.51), mild (0.43 to 0.51), moderate (0.26 to 0.43) and with severe alveolar bone loss (< 0.26) based on the quartile of the averaged proportion. The third molars were excluded from the count due to their complex shapes. Dentures, partial dentures, and complete implant bridges in either jaw were not included when determining the number of remaining teeth [16].

### Mortality followup

An annual review of mortality was conducted by Shanghai Municipal Center for Disease Control and Prevention, which collected mortality information for all the permanent residents in Shanghai. Death certificates in Shanghai are completed either by community doctors for deaths at home or by hospital doctors for deaths in hospitals. The information on the certificates was coded according to the International Classification of Diseases, Revision 10 (ICD 10). We defined total cancer as C00-99, LOP cancer as C00-14, digestive cancer as C15-26 and respiratory cancer as C30-39.

### Measurement of comorbid conditions

Information on comorbid conditions was collected from the Shanghai Health Information Center, which is responsible for maintaining records of outpatient visits, hospital admissions, and discharge for all hospitals in Shanghai. Body mass index (BMI) was determined by dividing weight (kilograms) by height (meters) squared (kg/m^2^). Participants with a BMI less than 25 were categorized as having a normal weight, while those with a BMI of 25 or higher were considered overweight. The education levels of the participants were grouped into three categories: those with six years or less of education, those with seven to twelve years of education, and those with university education. In our study, the smoking status was classified as non-smoker, previous smoker, or current smoker and drinking status as never drinking, sometimes drinking, or drink every day.

### Statistical analysis

Data were presented as numbers and percentages for categorical variables, and continuous data were expressed as mean ± standard deviation (SD) unless otherwise specified. We computed the person-time of follow-up for each participant from the date of panoramic radiography to the date of cancer mortality or the end of follow-up (December 31, 2021), whichever occurred first. Multivariate adjusted Cox proportional hazards model was fitted to control for the confounding effects of potential risk factors, including age, sex, BMI, education level, smoking, alcohol consumption, and the presence of comorbidities. HR and 95% CI were used to represent the effect of alveolar bone loss or tooth loss on the risk of cancer mortality. In the analysis, alveolar bone loss was treated as a continuous and categorical variable respectively to calculated HR (95% CI).

The standard Cox proportional hazards model is unsuitable for identifying risk factors associated with the cumulative incidence of specific events in the presence of competing risks. As a result, competing risk hazard models based on Fine and Gray were fitted for total cancer, LOP cancer, digestive cancer, and respiratory cancer mortality. In these models, mortality from the above diseases was set as the primary event of interest, and death from other causes was treated as a competing event.

All statistical analyses were conducted in R software, version 4.0.3 (R Development Core Team 2010).

## Results

We documented 104 total cancers, 28 LOP cancers, 39 digestive cancers and 13 respiratory cancers, over 10 years of follow-up. The characteristics of the subjects are presented in Table 1, both overall and by cancer site. Generally, cancer survivors have fewer missing teeth and less bone loss than those of non-survivors. The subjects who died of digestive cancer tended to have diabetes. Subjects who died of LOP cancer were less likely to have hypertension, while those who died of respiratory cancer were more likely to be male and nondrinkers.

**Table 1:** Baseline characteristics of study participants*

	Survivors	Total cancer	LOP cancer	Digestive cancer	Respiratory cancer
Participants	727(63.44%)	104(9.08%)	28(2.44%)	39(3.40%)	13(1.13%)
Residual alveolar bone%	0.39 (0.18)	0.33 (0.20)	0.314 (0.18)	0.35 (0.20)	0.30 (0.24)
Loss of teeth	13.50 (8.10)	15.60 (8.91)	15.40 (9.43)	15.40 (8.22)	16.20 (10.30)
Age	80.00 (3.39)	81.40 (4.54)	81.40 (5.21)	81.30 (4.42)	81.10 (5.06)
Sex					
Male	329 (45.30%)	53 (51.00%)	14 (50.00%)	19 (48.70%)	9 (69.20%)
Female	398 (54.70%)	51 (49.00%)	14 (50.00%)	20 (51.30%)	4 (30.80%)
BMI(kg/m^2^)					
< 25	579(81.32%)	79(81.44%)	19(79.17%)	33(89.19%)	8(61.54%)
≥ 25	133(18.68%)	18(18.56%)	5(20.83%)	4(10.81%)	5(38.46%)
Education levels(year)					
≤ 6	295(50.95%)	44(47.83%)	15(55.56%)	14(42.42%)	7(63.64%)
7–12	235(40.59%)	33(35.87%)	10(37.04%)	13(39.39%)	2(18.18%)
> 12	49(8.46%)	15(16.3%)	2(7.41%)	6(18.18%)	2(18.18%)
Smoking habits					
Nonsmoker	619(86.82%)	76(77.55%)	19(76.00%)	29(78.38%)	9(69.23%)
Previous smoker	77(10.80%)	17(17.35%)	5(20.00%)	6(16.22%)	3(23.08%)
Current smoker	17(2.38%)	5(5.10%)	1(4.00%)	2(5.41%)	1(7.69%)
Drinking habits					
Never drink	586(82.19%)	74(75.51%)	17(68.00%)	30(81.08%)	12(92.31%)
Drink sometimes	115(16.13%)	24(24.49%)	8(32.00%)	7(18.92%)	1(7.69%)
Drink everyday	12(1.68%)	0(0.00%)	0(0.00%)	0(0.00%)	0(0.00%)
Hypertension					
Without	158(21.73%)	42(40.38%)	18(64.29%)	12(30.77%)	5(38.46%)
With	569(78.27%)	62(59.62%)	10(35.71%)	27(69.23%)	8(61.54%)
Diabetes					
Without	676(92.98%)	95(91.35%)	27(96.43%)	34(87.18%)	12(92.31%)
With	51(7.02%)	9(8.65%)	1(3.57%)	5(12.82%)	1(7.69%)

*Age, loss of teeth and residual alveolar bone% are presented as mean(SD). The other variables are presented as number (%)

A significant increase in total cancer risk was associated with alveolar bone loss (HR 1.27, 95% CI 1.06–1.53). In addition, a borderline significant association was observed with LOP cancer (HR 1.45, 95% CI 0.99–2.12, P = 0.054). Furthermore, tooth loss was associated with a significant increase in cancer-related mortality. HR and 95% CI for mortality from total cancer was 1.03(1.00, 1.05). In the current study, neither alveolar bone loss nor tooth loss was associated with digestive or respiratory cancers (Table 2).

**Table 2:** HR and 95% confidence interval for cancer mortality^a^

	Quartile of residual alveolar bone%											Loss of teeth
	Q1(≥ 0.51)		Q2(0.43–0.51)		Q3(0.26–0.43)		Q4(< 0.26)		HR(95% CI) for trend^c^			HR(95% CI)
	HR(95% CI)		HR(95% CI)		HR(95% CI)		HR(95% CI)					
Total cancer	(n = 24)		(n = 17)		(n = 23)		(n = 40)					
Crude	1.00		0.75(0.40,1.40)		1.08(0.61,1.92)		1.93(1.17,3.21)		1.29(1.09,1.54)			1.03(1.01,1.06)
Adjusted^a^	1.00		0.73(0.38,1.40)		0.99(0.55,1.80)		1.86(1.11,3.14)		1.27(1.06,1.53)			1.03(1.00,1.05)
P value^b^			0.346		0.979		0.019		0.009			0.015
LOP cancer	(n = 3)		(n = 6)		(n = 9)		(n = 10)					
Crude	1.00		2.14(0.54,8.57)		3.31(0.90,12.26)		3.66(1.01,13.34)		1.46(1.04,2.07)			1.03(0.98,1.07)
Adjusted^a^	1.00		2.88(0.56,14.97)		4.77(1.02,22.35)		4.14(0.87,19.57)		1.45(0.99,2.12)			1.02(0.97,1.07)
P value^b^			0.208		0.047		0.073		0.054			0.381
Digestive cancer	(n = 11)		(n = 5)		(n = 7)		(n = 16)					
Crude	1.00		0.49(0.17,1.40)		0.74(0.29,1.91)		1.75(0.81,3.79)		1.27(0.96,1.69)			1.03(0.99,1.07)
Adjusted^a^	1.00		0.50(0.17,1.46)		0.53(0.18,1.55)		1.76(0.81,3.82)		1.25(0.94,1.68)			1.03(0.99,1.07)
P value^b^			0.206		0.249		0.153		0.130			0.170
Respiratory cancer	(n = 4)		(n = 0)		(n = 3)		(n = 6)					
Crude	1.00		0.00(0.00,999.99)		0.89(0.20,4.02)		1.83(0.52,6.53)		1.42(0.86,2.33)			1.04(0.98,1.11)
Adjusted^a^	1.00		0.00(0.00,999.99)		0.79(0.17,3.60)		1.61(0.45,5.85)		1.33(0.80,2.20)			1.04(0.97,1.11)
P value^b^			0.997		0.758		0.466		0.274			0.296

^a^Adjusting for age, sex, education levels, BMI, smoking, drinking, hypertension and diabetes

^b^ P-value from multivariate adjusted Cox proportional hazards model

^c^ HR(95% CI) for trend was calculated when severity of alveolar bone loss was treated as continuous variable

**Fig. 1:**
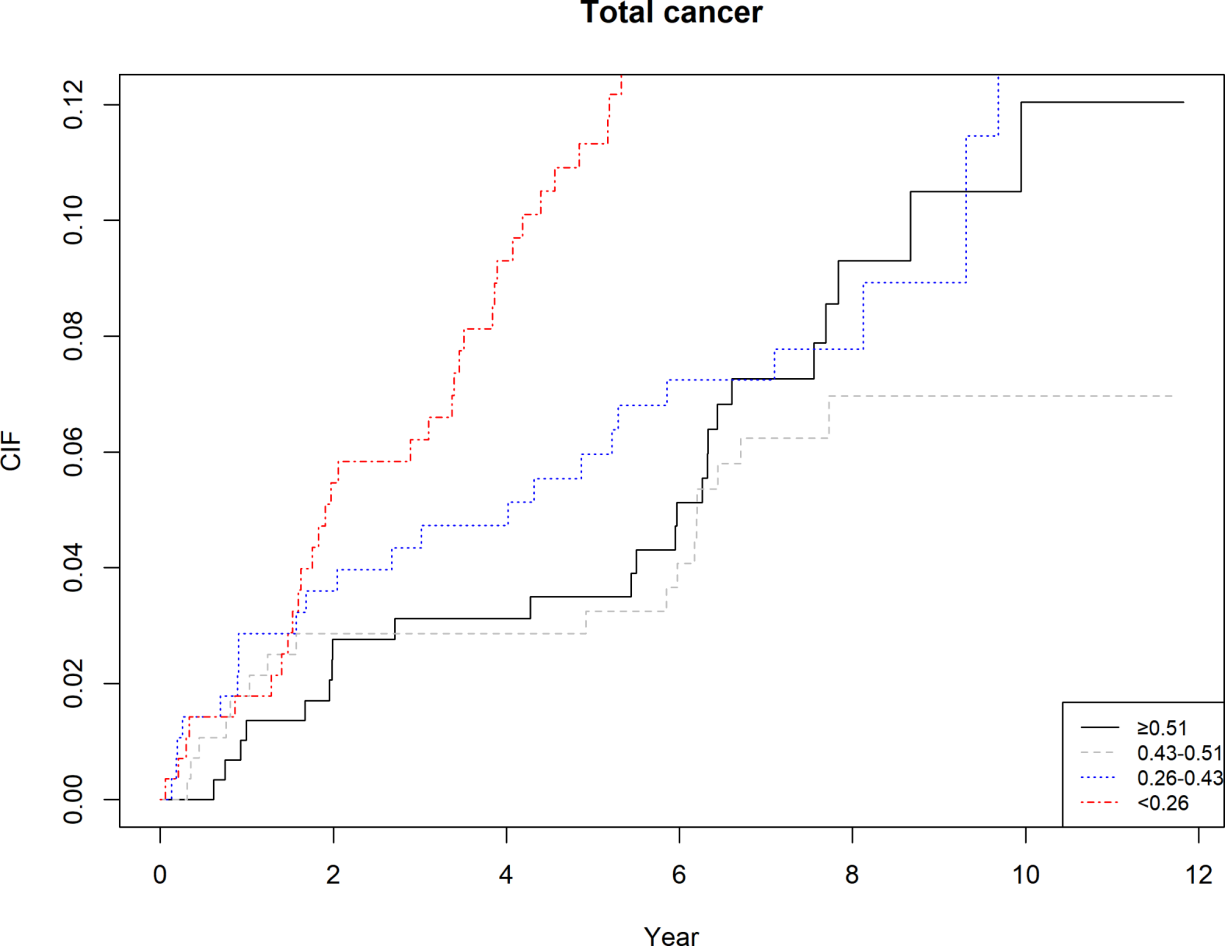
Increased risk of total cancer mortality associated with alveolar bone loss

**Fig. 2:**
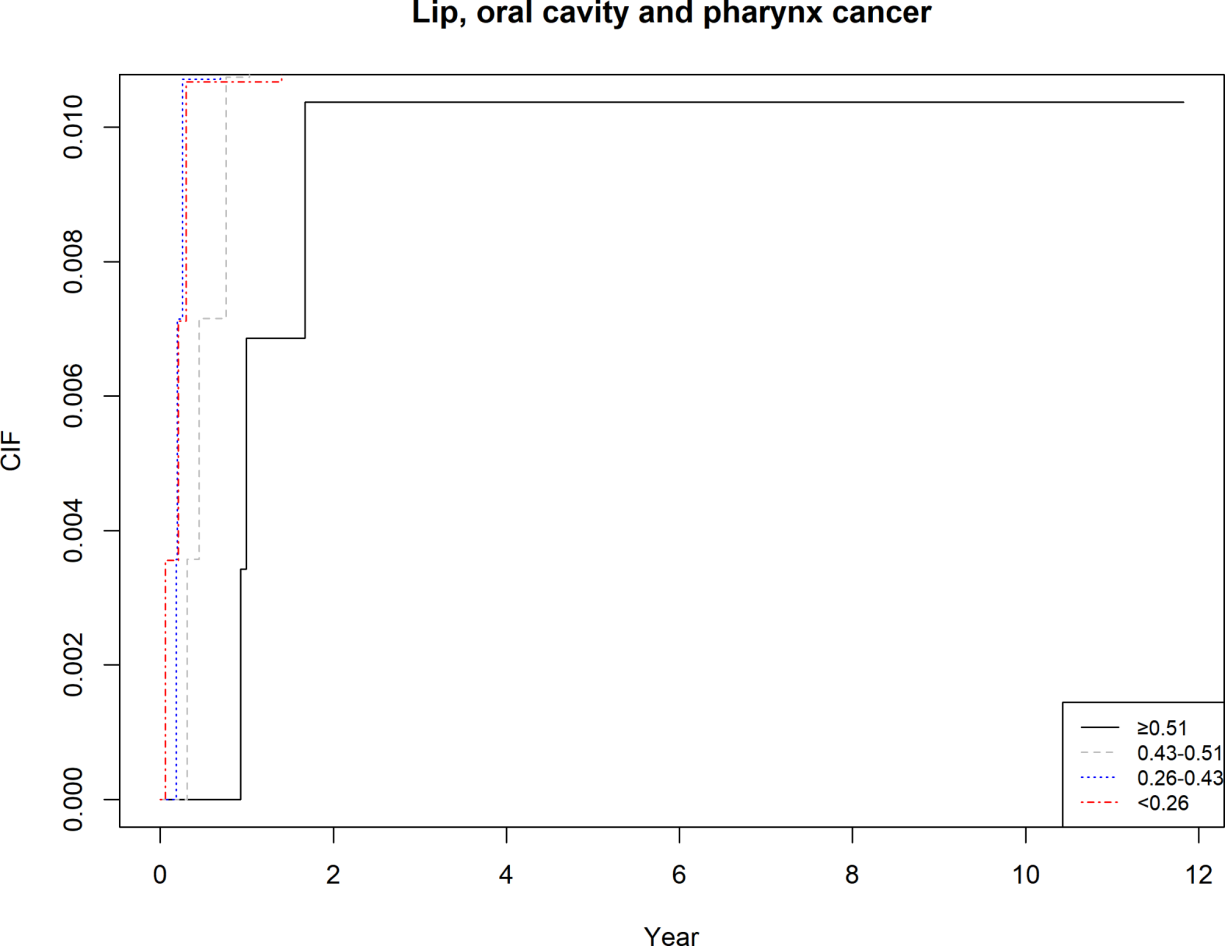
Increased risk of LOP cancer mortality associated with alveolar bone loss

As shown in Table 3, results from the multivariate competing risk hazard models suggested that alveolar bone loss was associated with a 28% and 45% increased risk of total cancer and LOP cancer mortality. Similarly, a 3% increase in the risk total cancer mortality was observed in the presence of tooth loss.(Figures 1 and 2 and supplementary Figs. 1–2).

**Table 3:** HR and 95% confidence interval of competing risk hazard models^a^

		Fine-Gray test	P value^b^	Adjusted HR (95% CI) ^c^	P value^d^
Total cancer	Alveolar bone loss	14.07	0.003	1.28(1.05,1.55)	0.015
	Loss of teeth	36.82	0.123	1.03(1.00,1.06)	0.021
LOP cancer	Alveolar bone loss	5.07	0.166	1.45(1.05,2.01)	0.025
	Loss of teeth	24.11	0.676	1.02(0.97,1.07)	0.380
Digestive cancer	Alveolar bone loss	8.66	0.034	1.25(0.89,1.77)	0.200
	Loss of teeth	39.96	0.067	1.03(0.99,1.07)	0.170
Respiratory cancer	Alveolar bone loss	6.35	0.096	1.33(0.75,2.34)	0.330
	Loss of teeth	30.97	0.318	1.04(0.96,1.11)	0.340

^a^Adjusting for age, sex, education levels, BMI, smoking, drinking, hypertension, and diabetes

^b^ P for Fine-Gray test ^c^ Severity of alveolar bone loss was treated as continuous variable ^d^ P for multivariate model

## Discussion

Characterized by chronic infection and inflammation, the prevalence of PD increases with age [17]. Tooth loss and alveolar bone loss, indicators of PD, have been found to be risk factors for the onset or death of many diseases. In this retrospective cohort study, we provided evidence that alveolar bone loss and tooth loss significantly increased the risk of total cancer and LOP cancer mortality in older patients, after adjusting for multiple potential confounders.

The number of teeth and self-reported oral health questions are commonly used to measure periodontitis in large cohort studies [12]. However, the extent of access to routine oral healthcare and the educational level may affect the effectiveness of self-reported periodontitis. Periodontitis may be misclassified and underestimated, thus weakening its association with cancer risk [10]. In our study, alveolar bone loss and loss of tooth were measured as indicators of periodontitis. Radiographic bone loss is one of the defining criteria for the classification of periodontitis. All the recruited patients in the current study were examined with panoramic radiographs, which allow us to assess alveolar bone loss in a detailed and precise manner. The primary causes of tooth loss are dental caries and periodontal disease, although the relative percentages of contribution from each condition vary depending on age and other factors. In younger individuals, dental caries is typically responsible for tooth loss, while chronic periodontal disease is more commonly associated with tooth loss in older individuals. Tooth loss is a better indicator of periodontal disease rather than dental caries in our study, since all the patients aged over 75.

Studies have found conflicting evidence for the link between periodontal disease and total and specific cancer. A cross-sectional study conducted by the U.S. Center for Disease Control and Prevention found that edentulism was associated with an increased risk of total cancer mortality [2]. Similar results were observed in cohort studies, with the increase in total cancer risk ranged between 14% and 20% [14, 18, 19]. However, in studies restricted to never smokers, positive results were only found in male, and no association was noted in a cohort of women [19]. Various studies have presented data on different measures of periodontal disease and their associations with oral cancer. Significant differences in plaque index, clinical attachment loss and radiographic bone loss was found between patients with oral cancer and controls [20, 21]. In contrast, Kerstin et al. reported that marginal bone loss was not associated with the presence of oropharyngeal squamous cell carcinoma (OPSCC) [22]. In studies involving individuals who never smoked, no link was found between periodontal disease and lung cancer [9]. However, a prospective cohort study revealed a positive association between periodontal disease and lung cancer among women with higher smoking intensity [23]. The large variation in study population, study design, periodontitis measures and definition of disease could be contributing factors to discrepancies between studies. Understanding the biological mechanisms underlying the associations are crucial in determining whether the relationship between periodontal disease and cancer risk is a direct effect or a result of shared genetic and/or environmental factors.

The contribution of periodontitis to cancer-related mortality remains uncertain. The toll-like receptors (TLRs) on oral epithelial cells are stimulated by *Fusobacterium nucleatum* and *Porphyromonas gingivalis*, which can promote tumor progression. The activation of TLR is associated with cellular proliferation and evasion of anti-tumoral immune responses. In addition, TLR-induced inflammation can interfere with DNA repair mechanisms or induce DNA mutations by producing free radicals and active intermediates that cause oxidative/nitrosative stress [24–26]. Particularly, oral cancer cells are dysregulated by *Fusobacterium nucleatum* and *Porphyromonas gingivalis*, which have been established as possible etiological agents [27, 28]. An unhealthy oral environment may cause overly aggressive immune functions, alter gut microbiota, and therefore be associated with digestive cancer development [29].

Several aspects of this study warrant further investigation. First of all, we did not perform clinical examinations. Periodontitis was measured based on alveolar bone loss and tooth loss. Neither the probing depth nor bleeding on probing, which may better represent periodontitis, were included in our analysis. Second, several potential confounders were controlled for in our study because of the well-characterized subjects. Nevertheless, there is still the possibility that the residual confounding of these factors may affect our results. Third, a relatively small number of cancer cases observed in our study may not have provided sufficient power to examine the outcomes, especially for digestive and respiratory cancer mortality. Finally, our study was biased by the retrospective nature of data collection. The results of this study still need further validation in the future studies.

## Conclusion

The findings of the present study suggest that older patients with periodontitis or tooth loss are at an increased risk of cancer mortality, especially for total cancer and LOP cancer. Intervention studies that include the treatment of periodontitis are warranted to determine whether the cancer risk can be reduced overall or at specific high-risk sites.

### Electronic supplementary material

Below is the link to the electronic supplementary material.

Supplementary Material 1

## Data Availability

The data that support the findings of this study are available from the corresponding author upon reasonable request.
