# Supplementary material for: Alveolar bone loss and tooth loss contribute to increase in cancer mortality among older patients
Source: BMC Oral Health. 2023 Dec 19;23:1023. doi: 10.1186/s12903-023-03543-5 (PMC10731843; doi:10.1186/s12903-023-03543-5)
Supplement: Supplementary file 1 — Supplementary Material 1 [file 12903_2023_3543_MOESM1_ESM.docx]

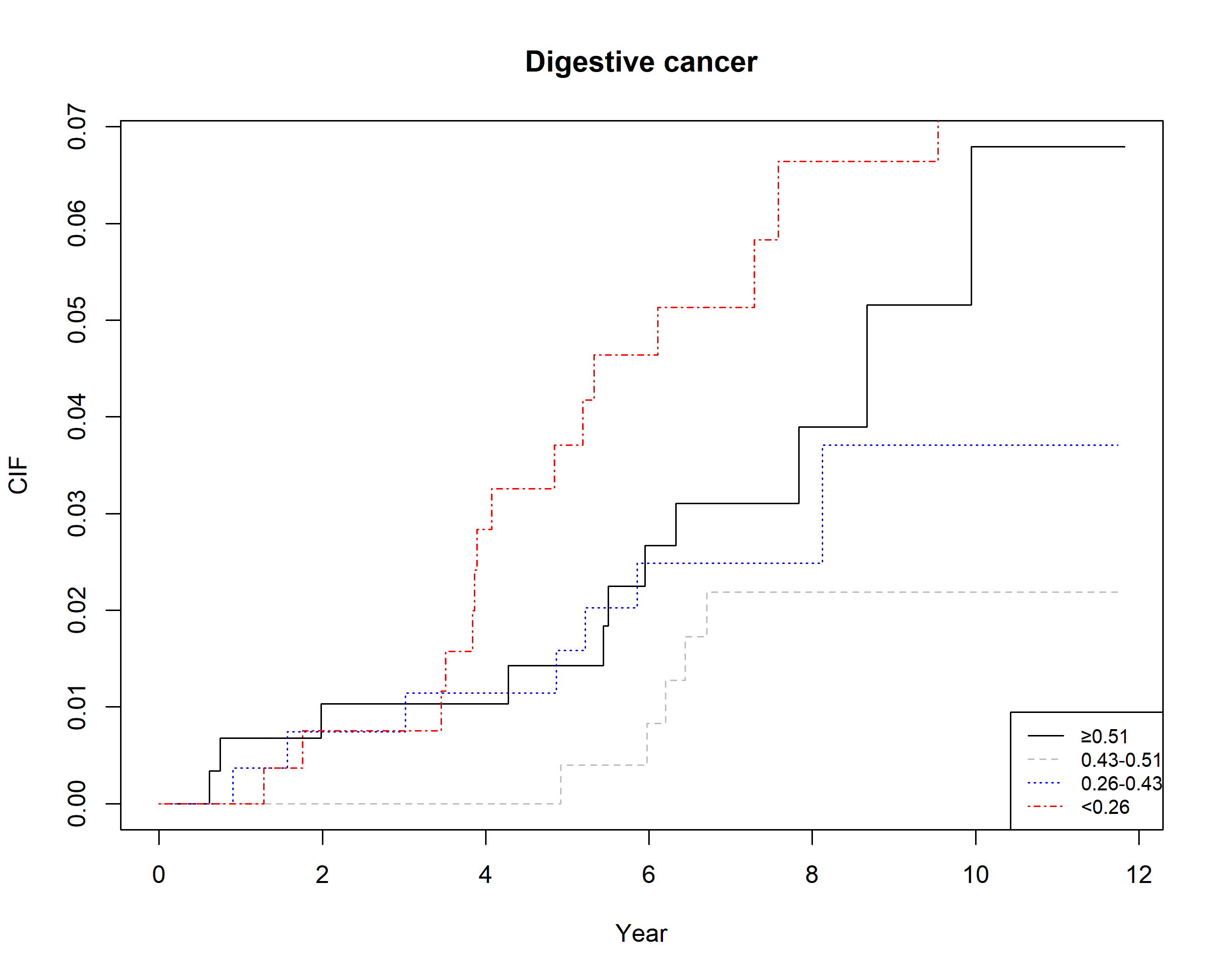


Supplementary figure 1. Increased risk of digestive cancer mortality associated with periodontal severity


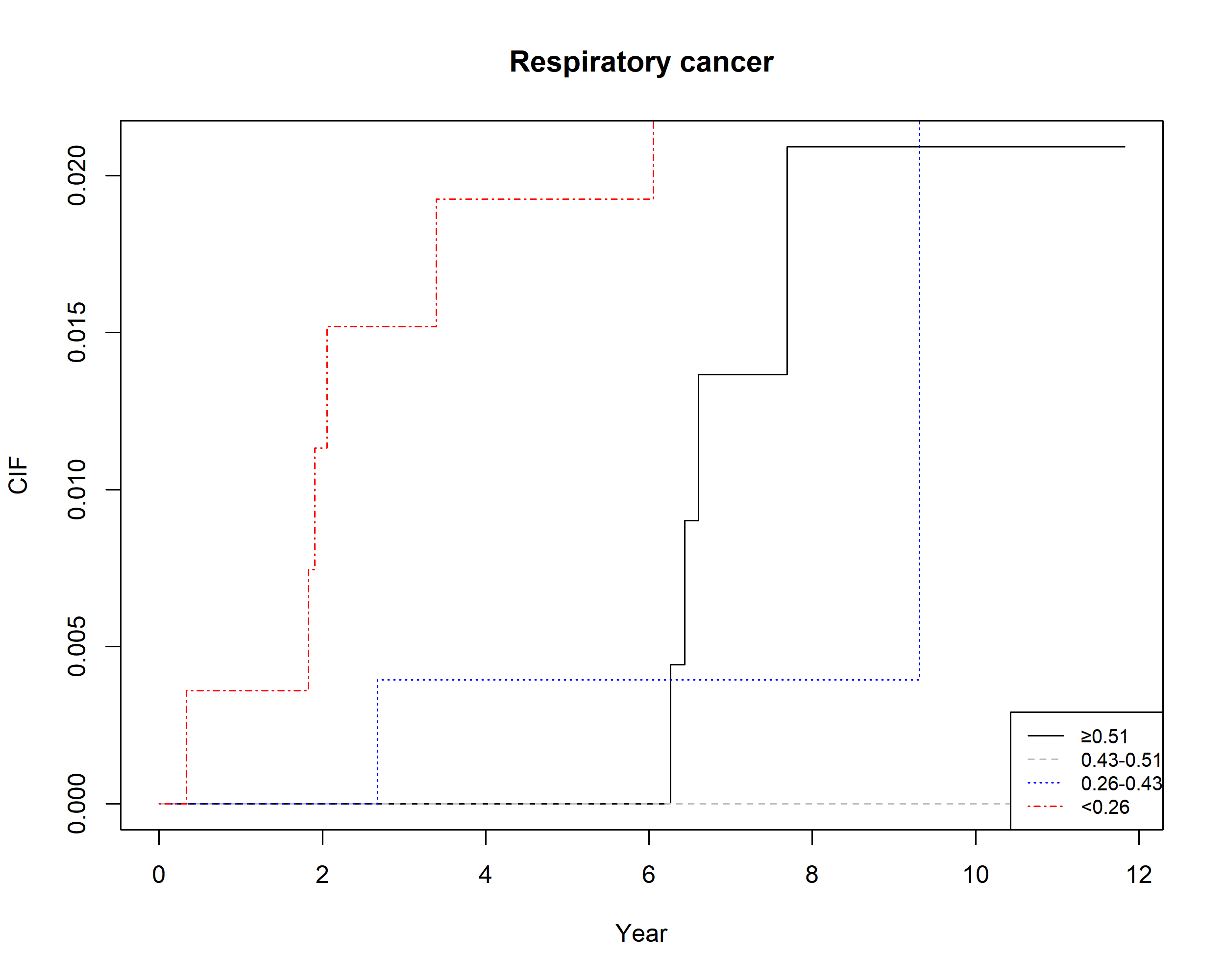


Supplementary figure 2. Increased risk of respiratory cancer mortality associated with periodontal severity
